# Insights into pet-based radiogenomics in oncology: an updated systematic review

**DOI:** 10.1007/s00259-025-07262-7

**Published:** 2025-04-07

**Authors:** Luca Filippi, Luca Urso, Luigi Manco, Michela Olivieri, Ilham Badrane, Laura Evangelista

**Affiliations:** 1 Department of Biomedicine and Prevention, University of Rome “Tor Vergata”, via Montpellier 1, Rome, 00133 Italy; 2 Department of Translational Medicine, University of Ferrara, Via Aldo Moro 8, Ferrara, 44124 Italy; 3 Medical Physics Unit, University Hospital of Ferrara, Ferrara, Italy; 4 Medical Physics Unit, IRCCS Humanitas Research Hospital, Rozzano, Italy; 5 Nuclear Medicine Unit, IRCCS Humanitas Research Hospital, Rozzano, Italy; 6 Department of Biomedical Sciences, Humanitas University, Pieve Emanuele, Milan, Italy

**Keywords:** Radiomics, Radiogenomics, Molecular imaging, Radiomic quality score, PET/CT, Cancer

## Abstract

**Purpose:**

This study systematically reviews current evidence on radiogenomics applied to positron emission tomography (PET) imaging across oncological diseases. The primary objective is to evaluate how PET-based radiogenomics aids in understanding tumor biology, prognostic stratification, and clinical outcome prediction, while identifying methodological challenges in the field.

**Methods:**

A systematic review was conducted following PRISMA guidelines, focusing on English-language studies indexed in Scopus, PubMed, and Web of Science until October 31, 2024. Inclusion criteria targeted original research articles involving human oncology studies using radiomics and genomics in a comprehensive “omics” framework. Data extraction included patient cohorts, radiopharmaceuticals and statistical methods. Studies were assessed for methodological rigor and reporting quality according to radiomics quality scores (RQS 2.0).

**Results:**

Eighteen studies involving 1780 patients were included, with 75.8% focused on lung cancer. Most studies were retrospective (72.2%) and single-center (77.7%). The primary radiopharmaceutical was [^18^F]FDG (88.8%). Key findings demonstrated correlations between PET-derived radiomic features and genomic alterations, such as KRAS, EGFR, and TGFβ mutations in lung cancer, and prognostic biomarkers in other malignancies. However, systemic shortcomings, including limited external validation, low reproducibility, and inadequate harmonization, were prevalent. None of the studies exceeded 50% of the RQS maximum score.

**Conclusion:**

PET-based radiogenomics holds significant potential for advancing precision oncology by capturing tumor heterogeneity and improving prognostic stratification. However, methodological limitations, particularly regarding study design and data transparency, hinder its clinical applicability. Future research should prioritize multicentric designs, robust external validations, and enhanced standardization to fully realize the discipline’s potential.

**Supplementary Information:**

The online version contains supplementary material available at 10.1007/s00259-025-07262-7.

## Introduction

The identification of tumor characteristics is essential to pursue precision oncology. Neoplasms are heterogeneous entities that require investigation through a multimodal diagnostic approach. In this context, the associations of “omics” modalities, such as genomics (laboratory tools) and radiomics (imaging) represents an innovative and potentially revolutionary way to investigate tumor heterogeneity and to enable patient-tailored treatment approaches [1].

Genomics implies the study of the whole genome, enabling extraction of a large amount of cancer-related genetic features [2]. This modern “omic” approach is allowed by next-generation sequencing (NGS), which is a modern DNA sequencing system enabling to decode large amounts of DNA in short time [3]. Radiomics seems to offer an almost unlimited supply of in vivo biomarkers which specifically are imaging biomarkers. Radiomics is a young discipline that has seen tremendous growth in recent decades in parallel with the development and innovations in medical image analysis. It is based on the high-throughput extraction (automated or semi-automated) of a multitude of quantitative features from digital medical images [4]. This process of extraction/conversion of biomedical images in quantitative parameters is motivated by the concept that such images contain additional information not visible to the human eye. Moreover, they reflect the underlying pathophysiology and more specifically, genomic and proteomic patterns that can be expressed in terms of image-based macroscopic features [5, 6, 7]. In the field of nuclear medicine, radiomic analysis of positron emission tomography (PET) is a hot topic. Most of the data currently available in the literature focus on [^18^F]Fluorodeoxyglucose ([^18^F]FDG) PET, which is widely used for diagnosis, staging, assessment of treatment response and survival prediction of several malignancies [8, 9, 10, 11]. Recently, PET imaging with other radiopharmaceuticals is also being investigated in terms of radiomic analysis, including radiolabeled prostate-specific membrane antigen (PSMA) in prostate cancer patients [12].

The ability to infer gene expression or mutation status – which can potentially contain useful information for prognostic stratification or to support therapeutic decision – from the quantitative analysis of medical imaging data is called radiogenomics [13]. This discipline is potentially applicable to any imaging technique. The extraction of a large amount of quantitative data through “omics” approaches requires the implementation of adequate processing tools. Among those, artificial intelligence (AI) plays a key role, enabling the training of prediction models that may be applied in daily clinical practice [14].

In this article we will systematically review the current literature evidence regarding radiogenomics applied to PET imaging in different oncological diseases.

## Materials and methods

### Search strategy

Following the PRISMA guidelines, our systematic review involved a comprehensive literature search of English-language articles listed in Scopus, PubMed, and Web of Science. No specific start date was set, and the search results were last updated on October 31, 2024. The search terms included “radiomic” and “genomic” or “radiogenomic” and “PET” or “positron emission tomography.” The search was conducted both with and without filters, such as language (English only), article type (original and research articles), field of interest (oncology only), and subject (human studies only). Reviews, clinical reports, conference abstracts, and editorials were excluded from consideration, as well as studies focused on single mutations or sets of mutations that did not adhere to the “-omics” approach (i.e., comprehensive DNA analysis, including all genes). Studies with patient cohorts of fewer than 10 were also excluded.

Two reviewers (L.U. and L.F.) conducted the literature search, selected studies for inclusion, and excluded duplicate articles. Any discrepancies were resolved by consensus. After merging all identified records, the full texts were retrieved and further assessed by three reviewers (L.U., L.F., and L.E.). Additionally, one reviewer (L.E.) conducted a secondary search across the databases and reviewed references from already-selected studies to confirm eligibility.

### Data extraction, quality assessment, and data analysis

For each study included, general data such as authors, publication year, country, study design, patient number, radiopharmaceutical used, disease, and study endpoint were collected. Technical data were also extracted, including texture analysis methods, the number and types of features, software used, and types of statistical analysis.

Selected imaging studies were assessed using a modified version of the Critical Appraisal Skills Programme (CASP) checklist for diagnostic test studies (https://casp-uk.net, accessed on 15 December 2024). Two reviewers (L.F. and L.E.) conducted the critical appraisal, resolving any discrepancies through discussion with other authors.

For each study, the radiomics methodology was evaluated using the radiomics quality score (RQS 2.0, available at https://www.radiomics.world/rqs2, accessed on 13 January 2025), a framework introduced by Lambin and colleagues in 2017 [15], designed to assess the quality of reporting in radiomics research. RQS 2.0 consists of 36 checkpoints that either reward or penalize radiomics studies to promote optimal scientific practices, with a maximum score of 66 (100%). For a robust evaluation, RQS 2.0 was independently calculated by two medical physicists (M.O. and L.M.), with any discrepancies discussed to reach a consensus.

## Results

**Fig. 1:**
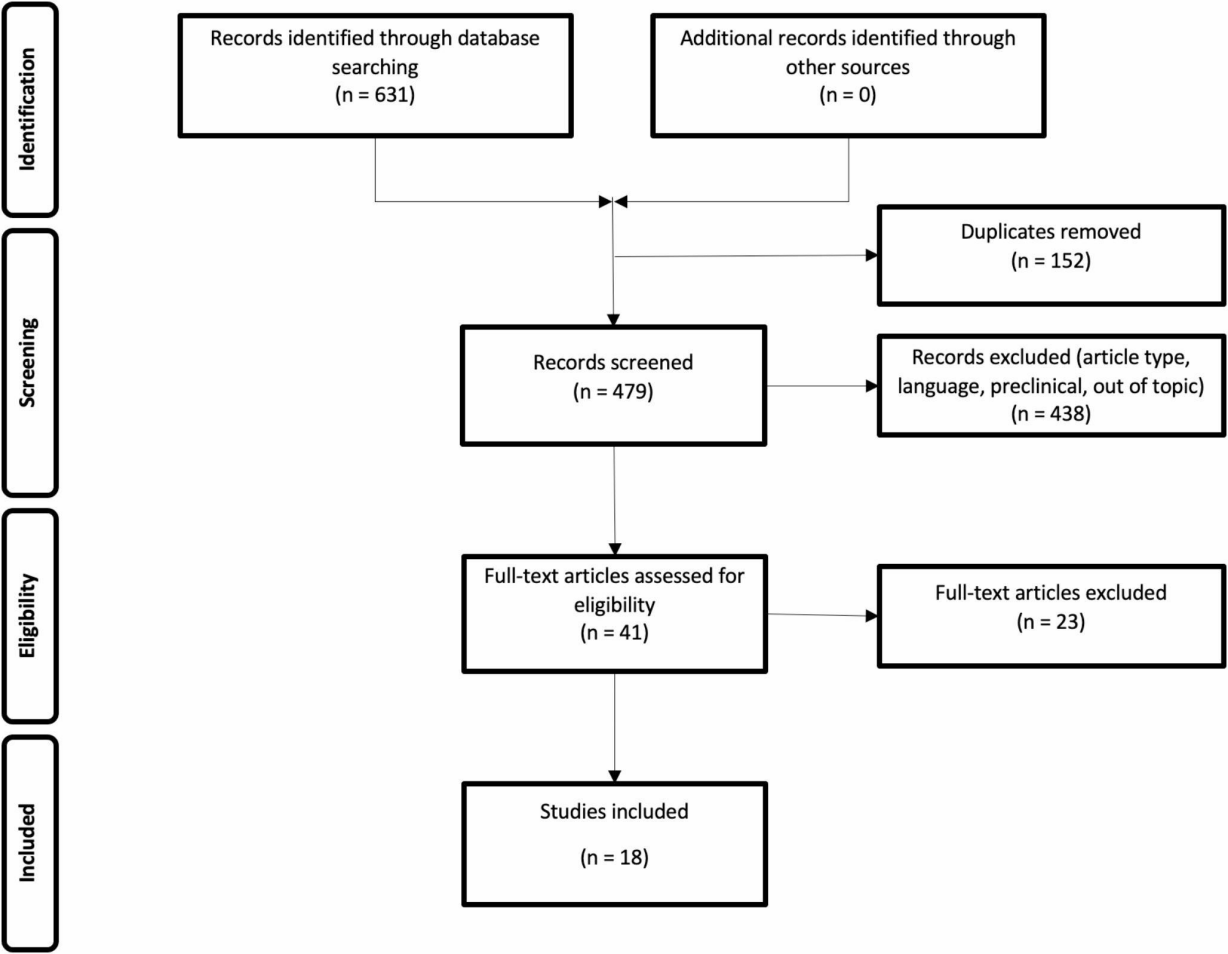
PRISMA flowchart indicating the selection process of the included studies

The resulting PRISMA search strategy is represented in Fig. 1. The analysis included 18 studies with a total of 1780 patients. Among these, 13 studies were retrospective, 2 were prospective, and 3 could not be classified as they relied on publicly available databases rather than their own patient cohorts. The results of the selected studies are summarized in Table 1.

**Table 1:** Clinical details of the studies included in the analysis

Authors	Year	Country	Study type	Setting	Number pts	RP	Disease	Endpoint
Nair et al. [31]	2012	U.S.	Multicentric	Retrospective	172	[18 F]FDG	NSCLC, predominantly adenocarcinoma	Survival prediction based on FDG uptake features
Gevaert et al. [15]	2012	U.S.	Single-center	Retrospective	26	[18 F]FDG	NSCLC, predominantly adenocarcinoma	Identification and prognostic validation of imaging biomarkers (e.g., tumor size, edge shape).
Bakr et al. [29]	2018	U.S	Single-center	Retrospective	211	[18 F]FDG	NSCLC, predominantly adenocarcinoma	Associations between imaging features, molecular profiles, and survival outcomes.
Kim et al. [16]	2020	Korea	Single-center	Retrospective	137	[18 F]FDG	NSCLC, predominantly adenocarcinoma	Association of PET features with genetic mutations and oncogenic signaling pathways.
Kirienko et al. [19]	2021	Italy	Single-center	Retrospective	74	[18 F]FDG	NSCLC, predominantly adenocarcinoma	Predicting histology and patient outcomes (recurrence). Identifying gene expression signatures associated with aggressiveness and relapse
Aide et al. [20]	2022	France	Single-center	Retrospective	109	[18 F]FDG	NSCLC, adenocarcinoma	Combining clinical and PET-derived radiomic features to predict the presence of molecular alterations in key molecular targets in lung adenocarcinoma.
Chen et al. [23]	2023	Taiwan	Single-center	Retrospective	46	[18 F]FDG	NSCLC, predominantly adenocarcinoma	To assess (a) the correlation of radiogenomic features with clinical data; (b) ICC of different genomic heterogeneity features; (c) ICC of PET-based heterogeneity features from different image matrix sizes.
Ju et al. [24]	2023	South Korea	Single-center	NA	53	[18 F]FDG	NSCLC, predominantly adenocarcinoma	To predict distant recurrence according to a model built on PET-derived radiomic features and gene expression.
Hinzpeter et al. [30]	2024	Canada	Single-center	Retrospective	128	[18 F]FDG	NSCLC, predominantly adenocarcinoma	To investigate the correlation between radiomics features extracted from [18 F]FDG PET/CT and driver gene mutations.
Sujit et al. [28]	2024	U.S.	Multi-center	Retrospective	394	[18 F]FDG	NSCLC, predominantly adenocarcinoma	Prognostic stratification of NSCLC patients according to radio-genomic analysis.
Ning et al. [27]	2024	Austria	Single-center	Prospective	146	[68Ga]Ga-PSMA-11	Prostate Cancer	To assess whole-mount Gleason Grade in prostate cancer by using ML model
Kesch et al. [26]	2018	Germany	Multi-center	Prospective	5	[68Ga]Ga-PSMA-11	Prostate Cancer	To define a genomic index lesion based on chromosomal copy number alterations as marker for tumor aggressiveness
Ferrer-Lores et al. [22]	2023	Spain	Single-center	Retrospective	33	[18 F]FDG	DLBCL	To assess the ability of FDG PET/CT features plus clinical data, alone or in combination with genomic parameters to predict complete response to first-line chemotherapy
Kim et al. [25]	2021	Korea	Single-center	Retrospective	52	[18 F]FDG	Pediatric osteosarcoma	To estimate prediction model using gene expression and image texture features
Lim et al. [18]	2020	Korea	Single-center	Retrospective	48	[18 F]FDG	Pancreatic ductal adenocarcinoma	To assess the correlation between gene mutations and PET-based radiomics
Choi et al. [32]	2019	Korea	Single-center	NA	25	[18 F]FDG	Head and neck cancer	To investigate how the genetic and metabolic heterogeneity features of the tumor are associated with head and neck squamous cell carcinoma.
Lee et al. [21]	2023	Korea	Single-center	Retrospective	91	[18 F]FDG	Stage IV colorectal cancer	To explore the ability of FDG PET/CT to predict the genetic alterations of stage IV colon rectal cancer
Vlachavas et al. [17]	2019	Greece and Germany	Multi-center	NA	30	[18 F]FDG	Colorectal cancer	To explore the integration of PET/CT features and genomic alteration for predicting colon-rectal lesions

DLBCL = diffuse large B-cell lymphoma; FDG = [^18^F]Fluorodeoxyglucose; ICC = intra-class correlation coefficient; ML = machine learning; NA = not available; NSCLC = non squamous cell lung cancer; PET/CT = Positron Emission Tomography / Computed Tomography; PSMA = Prostate-specific Membrane Antigen; TMB = tumor mutation burden

According to CASP analysis, studies consistently demonstrated high methodological rigor in areas such as the clarity of research aims, appropriateness of qualitative methodologies, recruitment strategies, and data collection methods. However, some variability is observed, particularly regarding the relationship between researchers and participants and ethical considerations. Despite these limitations, the overall appraisal highlights that most studies were considered as “highly valuable” and received a positive summary. Notably, a subset of studies (*n* = 5) achieved particularly robust evaluations underscoring their methodological strength and the reliability of their findings (Supplemental material: Table S1).

**Fig. 2:**
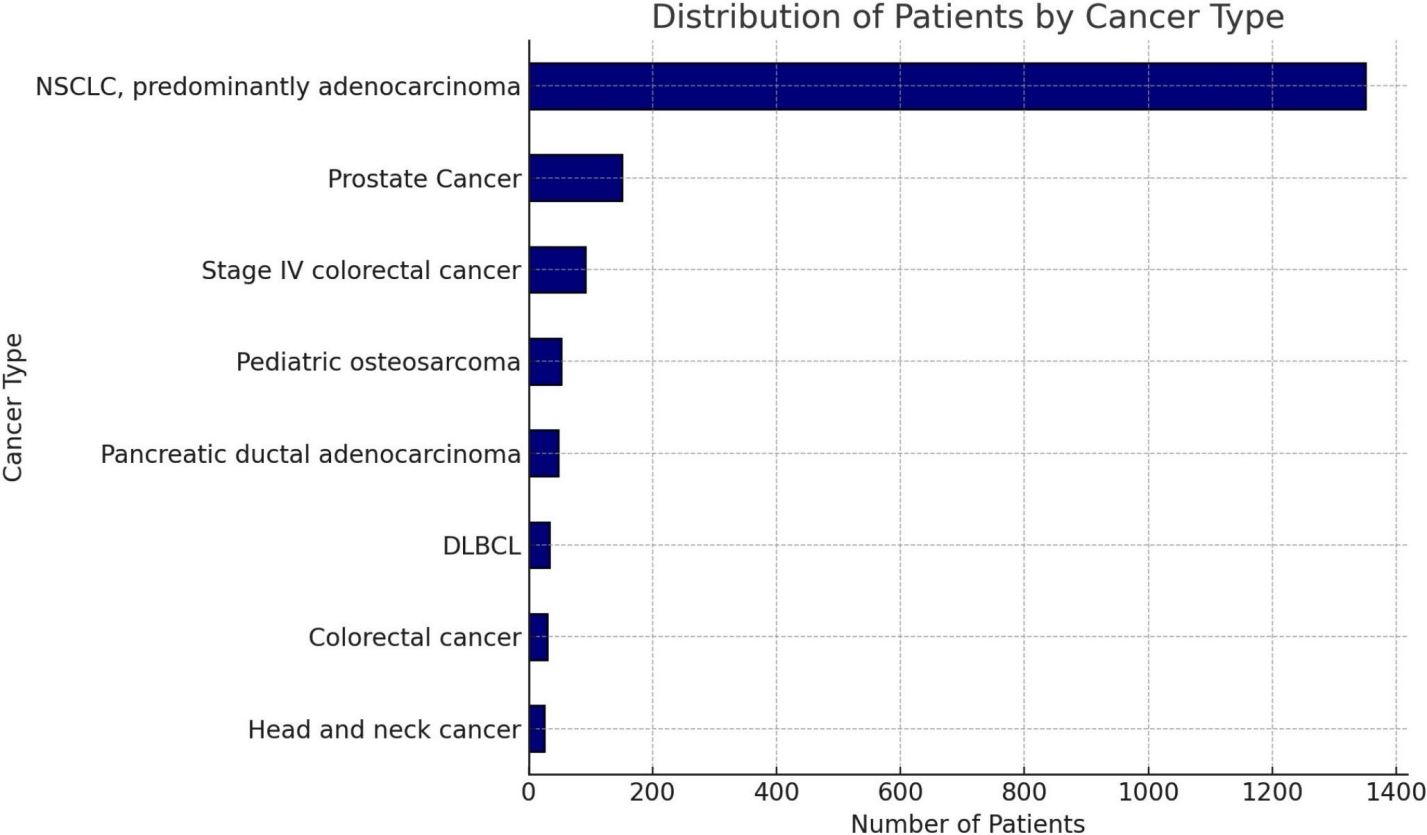
Distribution of the patient population according to the cancer type

The studies were divided into two main thematic areas: PET-based radiogenomics in lung cancer and radiogenomics in other cancers. The first thematic area, focused on lung cancer, included 10 studies with 1350 patients, accounting for 75.8% of the total cohort. The second area, which encompassed radiogenomics in other tumors, comprised 8 studies involving 430 patients, representing 24.2% of the total population. Specifically, this group included 2 studies on colorectal cancer, 2 on prostate cancer, and the remaining 4 addressing diverse cancer types such as diffuse large B-cell lymphoma, pediatric osteosarcoma, pancreatic ductal adenocarcinoma, and head and neck cancer. The distribution of the patient population stratified by tumor type is represented in Fig. 2.

Regarding radiopharmaceuticals, [^18^F]FDG was used in 16 out of 18 studies, representing 88.8% of the total. In contrast, 2 studies employed [^68^Ga]Ga-PSMA-11 for prostate cancer.

The studies were also characterized by variation in their geographic and institutional contexts. Fourteen studies (77.7%) were single-center, while six (33.3%) were multicentric. Geographically, the studies spanned several continents, most of the selected studies (8/18) in Asia. The temporal distribution of the studies ranged from 2012 to 2024, highlighting a consistent and increasing interest in radiogenomics over the years.

### RQS

**Fig. 3:**
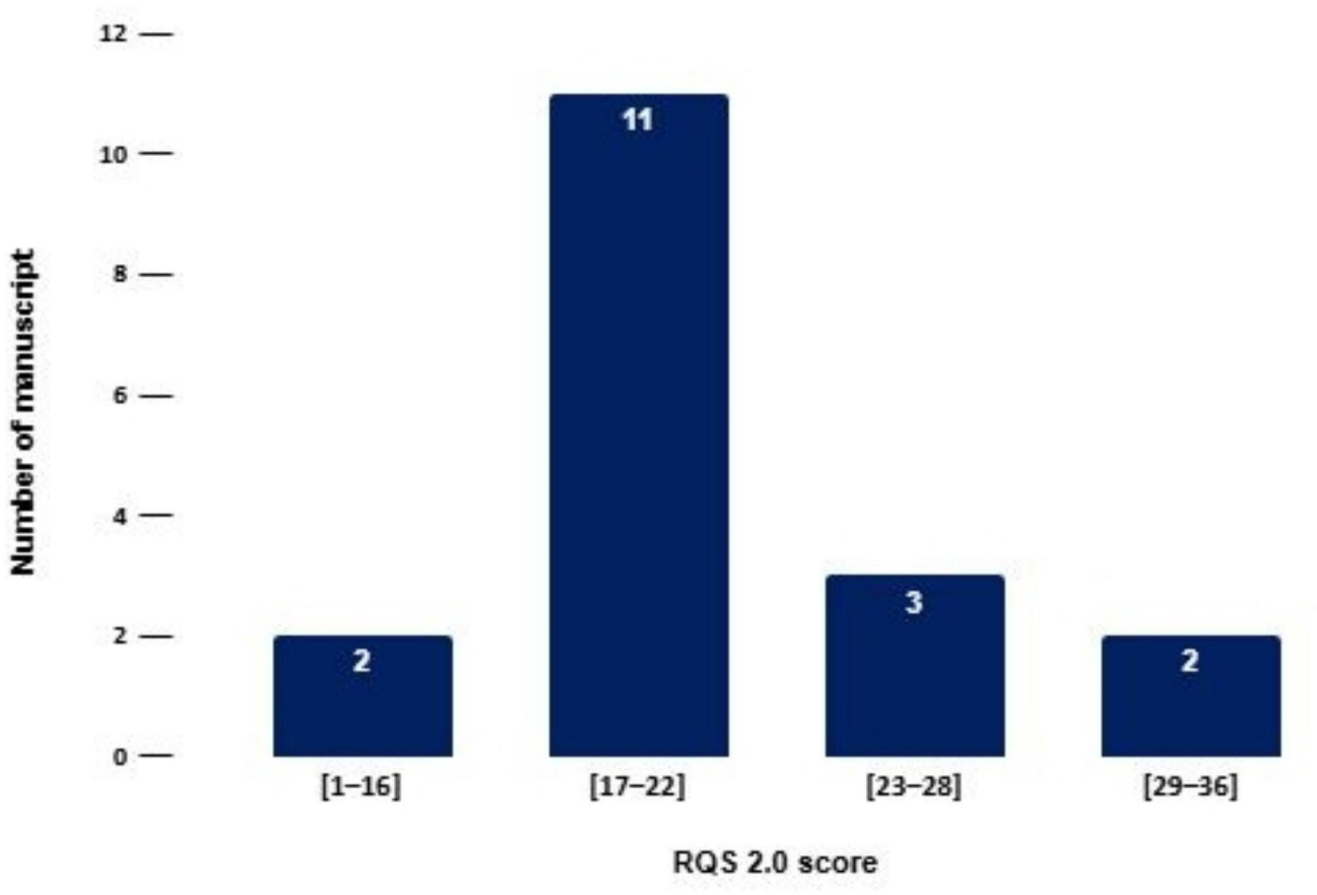
Results of the RQS analysis

The RQS 2.0 scores of the analyzed studies reveal systemic methodological shortcomings (Table 2, Table S2). None of the studies exceed 50% of the maximum RQS score (66). As highlighted in the provided histogram (Fig. 3), 61% of the analyzed studies fall within the range of 17–22 points [16, 17, 18, 19, 20, 21, 22, 23, 24, 25, 26]. Key areas with consistently low scores include prospective study design, external validation, data harmonization, transparency, and reproducibility. These limitations reflect fundamental challenges in the current state of radiomics research included in this study.

**Table 2:** RQS of the studies included in the analysis

Authors	Segmentation method (algorithm)	Segmentation SW (class)	FTs type (*n*)	Selected FTs	Radiomic SW (class)	Statistical analysis to reduce redundant variables (SW)	RQS (%)
Nair et al. [31]	Automatic (region-growing)	RT_Image (OS)	PET quantitative features (11) first order (3)	14	RT_Image (OS)	Spearman-rank correlation test (MATLAB)	25 (37.88%)
Gevaert et al. [15]	Manual	N.A.	computational (153) (histogram, texture, shape), semantic (26) (necrosis, nodules), SUV (1)	114 (CT) + 1 (PET)	N.A.	Lasso Regularization (R)	19 (28.79%)
Bakr et al. [29]	Automatic + manual (revision)	ePAD (OS)	Morphologic (45)	N.A.	N.A.	N.A.	11 (16.67%)
Kim et al. [16]	Semi-automatic (PET-edge tool)	MIM Software v.6.4 (C)	first-order, higher order GLCM, GLRM, NGLDM, GLSZM (86)	27	CGITA/MATLAB (OS/C)	T-test, Wilcoxon rank-sum test, clustering, Pearson correlation, ANOVA, Welch’s ANOVA or Kruskal Wallis, Kaplan-Meier, log-rank test, Cox (SAS v.9.4 and R v.3.5.3)	17 (25.76%)
Kirienko et al. [19]	Automatic (threshold-based and K-means clustering for PET and region-growing for CT)	3D Slicer	60 PET 57 CT	4	MATLAB (C) 3D Slicer (OS)	GLM (R v.3.6.1)	21 (31.82%)
Aide et al. [20]	Semi-automatic (PET-edge tool)	MIM software v. 5.6.5 (C)	first-order, shape, higher order GLCM, NGLDM, GLZLM (31)	6	LifeX v.6.30 (OS)	Mann Whitney, Lasso, ROC, Spearman correlation (XLSTAT v.2019)	18 (27.27%)
Chen et al. [23]	Semi-automatic (threshold method)	LifeX v.5.2.0 (OS)	first order (SUV, TLG) (5)	2	LifeX v.5.2.0 (OS)	Chi-square, T-test, Wilcoxon rank sum, univariate analysis, Lasso, Logistic Regression, ROC, AUC, (R v.4.3.0)	18 (27.27%)
Ju et al. [24]	Semi-automatic (threshold method)	LifeX v.4.0 (OS)	first-order, shape, higher order GLCM, NGLDM, GLSZM, GLRLM (47)	4	LifeX v.4.0 (OS)	Pearson correlation, T-test, AUC (R)	20 (30.30%)
Hinzpeter et al. [30]	Semi-automatic (threshold method)	LifeX v.6.30 (OS)	first order textural (GLCM, GLZLM, LZLGE, ZLNU, GLRLM, HGRE, NGLDM), shape (91 for CT and 96 for PET)	13 and 8 (CT) 13 and 17 (PET) 14 and 17 (PET/CT) for Model 1 and Model 2 respectively	LifeX v.6.30 (OS)	Spearman Correlation (R)	23 (34.85%)
Sujit et al. [28]	Manual	MIM Software (C)	first order (40) second order (4) MSI matrix (92)	98	MATLAB	Pearson’s Chi-square, univariate and multivariate analysis Cox proportional hazards (R v4.1.2)	30 (45.45%)
Ning et al. [27]	Manual	N.A.	first-order, shape, higher order GLCM, GLSZM, GLRLM, GLDM, NGTDM (107)	43, 2	PyRadiomics 3.0.1 (OS)	Mann-Whitney, Chi-square test (Python 3 package-scipy package 1.11.4)	28 (42.42%)
Kesch et al. [26]	Manual	MITK (OS)	first-order (21) texture (315)	1910	MITK (OS)	Variance test (R v. 3.3.0 and 3.4.2)	16 (24.24%)
Ferrer-Lores et al. [22]	Manual	Quibim Precision v2.8 (C)	first order (5), second order (GLCM, GLRLM, GLSZM, NGTDM)	17	Quibim Precision	Mann Whitney Wilcoxon, Logistic regression (Python v.3.8.12, R v.4.2.0 and RStudio)	21 (31.82%)
Kim et al. [25]	Manual	LifeX v.4.0 (OS)	PET quantitative features first-order second-order high-order (47)	7 + 17	LifeX v.4.0 (OS)	AUC, AUC_max (N.A.)	21 (31.82%)
Lim et al. [18]	Semiautomatic (gradient-based)	MiM Maestro (C)	first-order (6) shape (2) higher order GLRLM, NGLDM, GLSZM (27)	8	CGITA/MATLAB (OS/C)	Mann-Withney U test, ROC, AUC (SPSS v.23.0)	20 (30.30%)
Choi et al. [32]	Semi-automatic (PET-edge tool)	MIM Software (C)	SUVmax, SUVpeak, TLG, MTV, entropy, COV (6)	6	LifeX (OS)	Spearman Correlation (R v. 3.4.4 and SPSS v.25)	23 (34.85%)
Lee et al. [21]	Semi-automatic (threshold method) + manual (revision)	LifeX v.7.1.13 (OS)	first-order, higher order GLCM, GLRLM, GLSZM, NGTDM (157)	10	LifeX v.7.1.13 (OS)	FDR, ROC, Pearson correlation, T-test, Cox, Logistic regression, Kaplan-Meier, log-rank test (R v.4.0.4)	21 (31.82%)
Vlachavas et al. [17]	Manual	PMOD (C)	Kinetic, SUV (8)	5	PMOD	Lasso, Kaplan-Meier, log-rank test, Cox, Spearman correlation, AUC, Multivariate analysis: Multiple Factor Analysis (R)	22 (33.33%)

AUC = Area Under the Curve; C = commercial; COV = coefficient of variation; ePAD:= Electronic Physician Annotation Device; FDR = False Discovery Rate; GLCM = Gray-Level Co-occurrence Matrix; GLM = Generalized Linear Model; GLRLM = Gray-Level Run Length Matrix; GLRM = gray level run-length matrix; GLSZM = Gray-Level Size Zone Matrix; GLZLM = Grey-Level Zone Length Matrix; HGRE = High Gray-Level Run Emphasis, ICC = Intra-Class Correlation; LASSO = least absolute shrinkage and selection operator; LDA = Linear Discriminant Analysis, LZLGE = Long-Zone Low Gray-Level Emphasis; MSI = Multiregional Spatial Interaction, NA = Not Available; NGLDM = Neighborhood Grey-Level Different Matrix, NGTDM = Neighboring Gray-Tone Difference Matrix; OS = Open Source; ROC = Receiver operating characteristic; SUV = standardized uptake value; SW = software; ZLNU = Zone Length Non-Uniformity

In terms of radiomic analysis, only two studies present a prospective design [27, 28], rather the retrospective approach is the dominant one. This methodological choice introduces significant bias and limits the robustness of the findings. Additionally, external validation is often missing or underdeveloped, as many studies rely on data from a single center, reducing the generalizability of the models. Data harmonization, an essential step to minimize variability introduced by different scanners and imaging protocols, is inadequately addressed in most studies. On the other hand, though, a specific image pre-processing pipeline to harmonize scans from different centers was developed by Sujit et al. [29]. Transparency is another critical issue, as raw data, metadata, and code are rarely shared, hindering reproducibility.

Further methodological issues include the lack of mitigation strategies to address biases in datasets, such as imbalanced class distributions or demographic variability, and insufficient analysis of cut-off values to stratify risk groups. The scarcity of phantom studies to assess and quantify scanner variability adds another layer of uncertainty, as differences in imaging hardware and settings can significantly affect radiomic features extraction.

Calibration statistics, a critical component for evaluating model predictions and ensuring their alignment with clinical outcomes, are frequently omitted. This omission reduces the credibility and utility of predictive models in practical applications. Furthermore, interpretability remains a significantly underexplored aspect of radiomics research. Most studies neglect to incorporate advanced methods such as SHAP (Shapley Additive Explanations), which are instrumental in linking radiomic features to clinically meaningful parameters. This limits their clinical applicability.

In particular, the studies by Bakr et al. and Kesch et al. [27, 30] exhibit the lowest RQS 2.0 scores. Both studies focus primarily on the conceptual development of radiomic models, with limited efforts toward robust validation or clinical integration. For example, Bakr et al. [30] introduce a radiogenomic dataset that combines imaging and molecular data but do not advance to applying this dataset in validated predictive models. Similarly, Kesch et al. [27] explore, in a prospective study, the correlation between genomic and imaging features in prostate cancer, emphasizing feasibility rather than clinical applicability.

In contrast, the study by Sujit et al. [29] achieves the highest RQS 2.0 score, setting a benchmark for methodological robustness. This study distinguishes itself by incorporating external validation across multiple centers and implementing advanced data harmonization techniques. The multi-institutional design, the external validation based on datasets from distinct institutes and focus on clinical applicability underscore its strength and potential impact on the field. The results of the RQS analysis are schematized in Fig. 3.

### Lung cancer

**Fig. 4:**
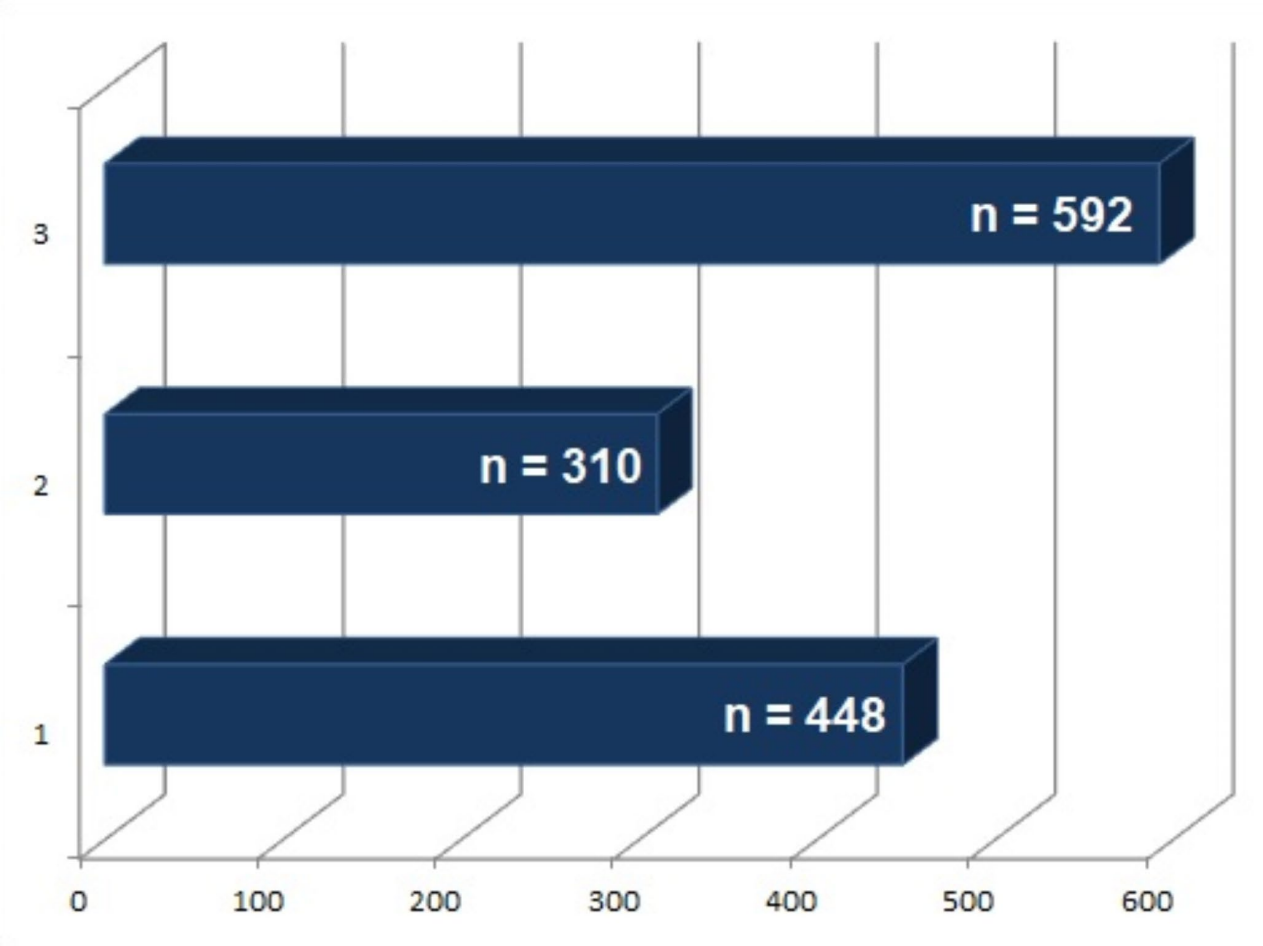
Distribution of the lung cancer patients according to the three macro-areas of radiogenomic analysis investigation: (**1**) correlation with driver gene mutations, or tumor biology; (**2**) prognostic stratification; (**3**) clinical outcome prediction

Several original studies used a radiogenomic approach to investigate different aspects in non-small cell lung cancer (NSCLC) patients. In particular, the main aim of the published papers could be summarized into three macro-areas: (a) Correlation with driver gene mutations, or tumor biology (*n* = 4); (b) prognostic stratification (*n* = 3); and (c) clinical outcome prediction (i.e., survival, *n* = 2; recurrence, *n* = 1). Lung cancer patient distribution into the three macro-areas is schematized in Fig. 4.

Kim and colleagues investigated the relationship between [^18^F]FDG PET-derived image features and genetic alterations in lung cancer [17]. The study included 137 patients with various histologies (adenocarcinoma, squamous cell carcinoma, and small cell lung cancer) who underwent [^18^F]FDG PET/CT imaging. Tumor lesions were segmented to extract 86 PET image features, and 381 genes were analyzed using an NGS-based targeted-sequencing platform (CancerSCAN). The analysis revealed correlations between specific PET features and genetic mutations, as well as oncogenic pathway alterations, including WNT, p53, and TGFβ signaling. Among the 24 PET features linked to genetic mutations, each was associated with between 1 and 7 genes. For adenocarcinoma, the most prevalent histology, 41 of the 381 target genes were significantly associated with PET image features. Notably, SUVpeak and SUVmax were linked to mutations in the TGFβ pathway in adenocarcinoma. SUVpeak, an indicator of metabolic intensity, was significantly higher in patients with TGFβ pathway mutations compared to those without. A similar experience was performed in the paper by Aide et al. [21]. The authors combined clinical (i.e. age, sex, smoking history, AJCC stage), and radiomic features extracted from baseline [^18^F]FDG PET to predict the presence of molecular alterations in a cohort of 109 patients with newly diagnosed lung adenocarcinoma. The authors performed an internal validation, and the presence of molecular alterations was defined according to NGS. Overall, the evidence of at least one molecular alteration was recognized in 57.8% patients, with KRAS and EGFR as the most frequently mutated genes (27% and 10%, respectively). The least absolute shrinkage and selection operator (LASSO) regression model built by the authors demonstrated a sensitivity and likelihood ratio of 90.0% and 1.35 to predict the presence of key molecular alterations at internal validation. In a cohort of 128 NSCLC patients, Hinzpeter et al. [31] investigated the correlation between radiomics features extracted from pre-surgical [^18^F]FDG PET/CT and driver gene mutations. Such a model would enable molecular profiling through non-invasive baseline imaging. However, the authors found only moderate prediction accuracy for driver gene mutations, including TP53, KRAS and EGFR. Thus, the radiomic model proposed by the authors is still far from the accuracy of NGS. Finally, in a retrospective, single-center observational study, Kirienko et al. analyzed data from 151 patients who underwent surgery for adenocarcinoma or squamous cell carcinoma and had baseline [^18^F]FDG PET/CT imaging [20]. A subset of 74 patients with cancer tissue samples available in the Institutional Biobank was included for radiomic and genomic analyses. The genomic analysis focused on identifying genetic variants, fusion transcripts, and gene expression profiles. ML identified robust predictive models, highlighting SUV and kurtosis (from PET and CT features, respectively) and the expression levels of TP63, EPHA10, FBN2, and IL1RAP as key tools for distinguishing adenocarcinoma from squamous cell carcinoma. While the ML approach modestly utilized PET radiomic features to predict relapses, it identified a strong gene expression signature capable of accurately predicting patient relapses. The top-performing radiogenomic rule for predicting relapse achieved an area under curve (AUC) of 0.87.

In a retrospective, multicentric study, Nair et al. investigated the prognostic value of [^18^F]FDG PET imaging in resected NSCLC [32]. Analyzing 25 tumor samples from the study cohort, the authors identified correlations between PET-derived features and gene expression signatures, as well as survival outcomes. Notably, a multivariate radiogenomic model incorporating [^18^F]FDG features (SUVmax, SUVvariance, and SUVPCA2) demonstrated significant predictive value in external (63 patients) and validation (84 patients) cohorts. The study also highlighted that certain PET-radiomic features reflect broader tumor bioenergetics and oncogenomic alterations, underscoring their potential as non-invasive biomarkers for improving NSCLC prognosis and understanding tumor biology. Following this approach, Gevaert et al. employed a radiogenomics strategy to identify prognostic imaging biomarkers for NSCLC [16]. By integrating imaging features from CT and PET/CT scans with gene expression data, the research established correlations between imaging characteristics and genetic profiles (metagenes). Conducted on a cohort of 26 patients, the study utilized public datasets to indirectly evaluate the prognostic significance of these biomarkers. Significant correlations were identified, highlighting tumor size, edge shape, and sharpness as the most relevant prognostic features. This innovative method has the potential to accelerate the translation of novel imaging biomarkers into personalized medicine without requiring extensive long-term clinical trials. To date, the study performing a radio-genomic analysis in the larger cohort of NSCLC patients (*n* = 394) was proposed by Sujit et al. [29]. The authors extracted 92 radiomic features from both diagnostic CT and [^18^F]FDG PET/CT and stratified newly diagnosed NSCLC patients into 3 imaging-based prognostic groups (low-, intermediate- and high-risk) that resulted significantly associated with ctDNA metrics. Moreover, a COX regression model combining the proposed imaging-based prognostic groups with clinical features, ctDNA metrics and tumor volume achieved a C-index = 0.82 to predict the risk of recurrence. Also, the authors performed a transcriptomic analysis from RNA sequencing that demonstrated that interferon α and interferon γ were downregulated in high-risk NSCLC groups. Although the authors define their work as a proof-of-concept study, the results proposed look more consistent than the average level of those published in the literature, as the results presented were validated both internally and externally in a rather large multicenter cohort protocol.

The study by Bakr and colleagues investigated a unique radiogenomic dataset of NSCLC, integrating imaging (CT, PET/CT), molecular, and clinical data from 211 patients [30]. This dataset included tumor segmentations, semantic annotations, and gene expression profiles obtained through microarrays and RNA sequencing. The study aimed to explore the relationships between tumor imaging features, molecular phenotypes, and survival outcomes. It demonstrated the potential of combining radiomic and genomic biomarkers to develop predictive tools for personalized treatment in NSCLC. Among the findings, the dataset highlighted significant associations between imaging features such as tumor size, texture, and [^18^F]FDG-PET uptake, with key oncogenic mutations (e.g., EGFR, KRAS, ALK) and survival outcomes. This underscored the value of non-invasive imaging biomarkers for capturing tumor heterogeneity and guiding precision medicine. The prediction of distant recurrence according to [^18^F]FDG PET-derived radiomic features and gene expression was the aim also of the paper by Ju and colleagues [25]. In 53 NSCLC patients, the authors extracted 47 radiomic features from PET images and correlated them with 37 gene modules identified by genomic analysis. A significant correlation was found between 4 radiomic features (GLRLM_SRHGE, GLRLM_HGRE, SUVmean, and GLZLM_GLNU) and 6 genes. The random forest (RF) Machine learning (ML) algorithm trained integrating both radiomic and genomic features reached an AUC of 0.912 to predict distant recurrence of NSCLC. In a cohort of 46 treatment-naïve NSCLC patients, Chen et al. [24] investigated radiogenomics from multiple points of view, including survival prediction. Firstly, they performed a technical evaluation, investigating the heterogeneity and reliability of both genomic and radiomic features according to the intra-class correlation coefficient (ICC) – a measure of consistency. Interestingly, they reported a moderate reliability (ICC = 0.736) for genomic features and a very high reliability (ICC > 0.9) for radiomic features, even though they were extracted from images obtained with different image matrix sizes. Afterwards, the authors found a significant shorter overall survival (OS) in patients with higher values of tumor mutation burden, SUVmax and entropy (extracted from reduced matrix-size images).

### Tumors other than lung cancer

From the eight papers analyzed, it emerged that radiomics by PET imaging can predict genetic mutations in prostate cancer [27], in pancreatic cancer [19], in head and neck cancer [33] and in colon rectal cancer [22]. Conversely, the association of omics, either radiomics by PET and genomics, in a computational model in patients affected by prostate cancer [28], lymphoma [23], pediatric osteosarcoma [26] and colorectal cancer [18] can be useful to improve diagnosis, predicting the response to therapy and playing a prognostic role.

In prostate cancer, radiomics by [^68^Ga]Ga-PSMA-11 PET/CT can predict the extent of genomic alteration defined as chromosomal copy number alterations (CNAs). However, the alone available study was an intentional preliminary analysis involving only 5 patients [27]. Machine learning analysis by including the association of radiomics by [^68^Ga]Ga-PSMA-11 PET/CT, pathomics and genomics in terms of tumor mutational burden (TMB) can predict the Gleason grade in a more accurate way, as compared to the biopsy, thus significantly affecting the risk stratification of prostate cancer patients [28].

In lymphoma patients, the identification of predictive variables able to identify patients with DLBCL who are responsive to a first-line immunochemotherapy is essential for better prognosis. However, the current strategies are not performing enough. The combination of gene rearrangements involving MYC, BCL2, and/or BCL6 and radiomic features from [^18^F]FDG PET/CT, such as GLSZM_Gray-LevelVarance and Sphericity can predict treatment response. When combined the AUC was equal to 0.904, thus opening the omics way in this setting of patients [23].

In pediatric patients with osteosarcoma, the identification of predictive variables is essential for improving the prognosis, that is still considered unfavorable for this neoplasm. A predictive model integrating radiomics by [^18^F]FDG PET/CT and gene expression can significantly identify patients with metastatic widespread and those who will exert a response to chemotherapy. Indeed, the combination of GLCM, EZRIN and ki76 can arrive at an AUC of 0.80 for the identification of metastasis. Whereas, the combination of NGLDM_Contrast, EZRIN and ki67 arrives at 0.89 for the prediction of response to chemotherapy [26].

In pancreatic cancer, radiomics features by [^18^F]FDG PET/CT can predict some genetic mutations such as KRAS and SMAD4 [19]. KRAS is associated with an uncontrolled proliferation property and with other processes. SMAD4 is a tumor suppressor protein. Both are expressed in pancreatic cancer, although not specific only for this neoplasm. Radiomics features based on voxel statistics or gray-level run-length matrix/size-zone matrix, such as SUV skewness, emphasis, can predict an AUC ranging between 0.692 and 0.829 gene mutations. Also, in head and neck cancer, radiomics features by [^18^F]FDG PET/CT can predict the tumor genetic heterogeneity [33]. Indeed, SUVmax, TLG, MTV, entropy and COV are predictors of MATH (mutant allele tumor heterogeneity) and GlycoS. In the study performed in 25 patients with stage III/IV head and neck cancer, the authors found that PET radiomics was associated with a mild degree to MATH that is a predictor of poor prognosis.

Finally, two studies are now available about radiogenomics in patients with colon-rectal cancer. The first study published in 2019 demonstrated the ability of gene expression in terms of RNA sequences and [^18^F]FDG PET/CT derived radiomics in making a differential diagnosis between benign and malignant colon-rectal lesions [18]. Later in 2023, [^18^F]FDG PET/CT derived radiomics features were found to be predictors of TMB in patients with stage IV colon-rectal cancer [22]. The authors underlined that PET radiomics were associated with tumor heterogeneity that can be linked to different gene expressions or to mixed immune cells composition.

## Discussion

Solid tumors are characterized by marked inter- and intra-tumoral genomic heterogeneity, and both spatial and temporal heterogeneity are observed in a single patient [34]. On the one hand, this encourages an increasingly personalized medical approach, but on the other it still represents the main cause of treatment failure, the onset of resistance to therapy and poor prognosis [35]. Therefore, there is a need to try to capture this heterogeneity to improve the treatment decision-making processes. However, biopsy-based profiling does not allow for adequate characterization of this heterogeneity. In fact, multiple biopsies over time would be needed (with all the consequences that an invasive maneuver entails) to capture temporal heterogeneity, but spatial heterogeneity would never be overcome since a biopsy is inevitably targeted to a single lesion and subject to sampling error. Similarly, imaging modalities allow visualization of tumor-related morphological or functional changes. However, they often lack cancer-specificity and allow representation of the oncological situation only at the few time-points when they are acquired.

Therefore, precision medicine research should aim not only at finding in vitro molecular biomarkers, but also at in vivo biomarkers spatially and temporally linked to tumor biology. The reviewed literature provides a fascinating, albeit preliminary, insight into the potential of PET-based radiogenomics in oncology. This field holds the promise of significantly enhancing our understanding of tumor biology, stratifying prognostic factors, and refining clinical decision-making. Lung cancer, in particular, serves as a prominent model for investigating mutation-driven therapies due to its high prevalence, genetic complexity, and established links between molecular alterations and therapeutic outcomes [11].

The importance of rapid and accurate genetic profiling in lung cancer cannot be overstated. Actionable mutations, such as EGFR, ALK, and KRAS, are critical for guiding targeted therapies, and delays in their identification can compromise patient outcomes [36]. Recent studies highlight the potential of liquid biopsy as a non-invasive alternative to tissue biopsy, enabling faster and safer mutation detection. However, liquid biopsy still faces significant challenges, including limited coverage of treatable mutations beyond EGFR and T790M, logistical barriers in processing samples within clinical timeframes, and the need for comprehensive analytical techniques [37, 38]. These limitations often result in delays in treatment initiation, underscoring the need for further advancements in molecular diagnostics and streamlined workflows. Furthermore, disparities in access to gene-driven therapies due to national reimbursement policies add another layer of complexity, restricting the full realization of precision oncology in many regions.

Against this backdrop, PET-based radiogenomics emerges as a promising tool to integrate molecular and imaging biomarkers. Lung cancer has been the focus of most studies in this domain, with 75.8% of the reviewed research addressing this malignancy. These studies have identified intriguing correlations between PET-derived radiomic features, such as SUVmax and SUVpeak, and key genetic alterations like KRAS, EGFR, and TP53 mutations. For instance, SUVpeak has been associated with TGFβ pathway mutations in adenocarcinoma, highlighting the potential of radiogenomics to capture tumor heterogeneity and inform therapeutic decisions [17]. However, while these findings are encouraging, they remain largely exploratory and far from clinical implementation. Moreover, severe limitations still affect the studies’ methodology. Thus, further efforts are required to enable the transition of radiogenomics into daily clinical practice.

Beyond lung cancer, the application of PET-based radiogenomics to other malignancies remains limited. While mutation-driven therapies have transformed treatment landscapes in cancers like melanoma [39, 40], radiogenomics has only been applied to a minority of oncological conditions. For example, preliminary studies in colorectal, prostate, and pancreatic cancers have shown promise in correlating PET-derived features with genetic alterations, but these efforts are hindered by small sample sizes, lack of external validation, and methodological inconsistencies [18, 19, 22, 22, 23, 26, 27, 28, 33]. Furthermore, in pediatric osteosarcoma and in lymphoma, radiogenomics would be useful to predict the responsiveness to therapy. In prostate cancer, the integration of radiomic, genomic, and pathomic data has been shown to improve risk stratification compared to biopsy alone. Similarly, in pancreatic cancer, radiomic features have demonstrated potential in predicting KRAS and SMAD4 mutations. However, these studies are still at an early stage, emphasizing the need for more robust investigations.

PET-based radiogenomics holds distinct advantages over CT or MRI-based radiogenomics due to its foundation in functional data that is intrinsically linked to the biological and behavioral aspects of tumors [41, 42]. Unlike anatomical imaging techniques, which provide static, structural information, PET imaging offers dynamic insights into metabolic activity, reflecting the tumor’s underlying physiology. This functional data is highly sensitive to changes in tumor biology, enabling a more accurate representation of its molecular characteristics. These advantages allow PET to provide critical information that is often missed by structural imaging modalities, making it an invaluable tool in understanding the tumor’s microenvironment and its response to therapies. In this regard, PET-based radiogenomics represents a groundbreaking approach for studying tumor heterogeneity at a genomic level in a precise and non-invasive manner [43]. By correlating the functional imaging data from PET scans with genetic and molecular profiles, researchers can uncover subtle variations in the tumor that may not be visible through other imaging methods. This capability opens up to new avenues for personalized treatment strategies, as it allows for a more detailed and comprehensive understanding of the tumor’s genetic landscape, ultimately contributing to more targeted and effective therapies. In an ideal approach, PET-based radiogenomics should integrate [^18^F]FDG imaging— despite its limited specificity—with molecular and genetic analyses, such as genomics and transcriptomics, which provide a more precise characterization of tumor biology. This combination would leverage the non-invasive nature of PET, capable of assessing tumor heterogeneity, with the specificity of omics-based analyses. However, integrating imaging and multi-omics data presents computational and logistical challenges, including data harmonization, standardization, and the need for advanced machine learning models [44]. Addressing these issues is essential to fully exploit radiogenomic signatures for patient stratification and personalized treatment.

Furthermore, radiogenomics can help identify gene signatures linked to tumor behavior by employing radiopharmaceuticals beyond [^18^F]FDG and PSMA ligands, such as FAPI, PD-1, and PD-L1 imaging. These agents provide insights into tumor microenvironment interactions and immune evasion, aiding in patient stratification for targeted therapies. FAPI imaging detects cancer-associated fibroblasts, while PD-1/PD-L1 imaging assesses immune checkpoint expression, guiding immunotherapy decisions [45, 46]. Though their integration with radiomics and gene expression remains unexplored, it holds great potential for advancing precision oncology.

All published PET-based radiogenomics studies have so far used static PET images due to the complexity and logistical challenges of dynamic PET, which requires prolonged scanning and often arterial blood sampling [47]. However, dynamic PET provides valuable tracer kinetic insights, potentially revealing additional tumor biology aspects. A study by Noortman et al. [48] found that while many dynamic radiomic features correlate with static ones, some dynamic features capture unique temporal information. Advances in PET technology, such as long axial field-of-view PET/CT scanners, now enable dynamic imaging with shorter scan times and non-invasive input function estimation [49, 50]. Future research should explore dynamic radiomics’ role in oncology and radiogenomics.

Radiomics methodology enables the extraction of numerous parameters, known as radiomic signatures, derived from image texture. However, the reproducibility of these signatures is influenced by various factors, including image acquisition, filtering, post-processing, segmentation strategies and feature extraction methods. To enhance consistency and reliability, the use of certified tools or standardized libraries is crucial. In this regard, PyRadiomics is a scientifically recognized standard. One of the most critical limitations in the current body of research lies in its methodological weaknesses. The predominance of retrospective and single-center studies (72.2% and 77.7%, respectively) introduces biases and limits the generalizability of findings. In this review, none of the studies surpassed 50% of the maximum RQS 2.0, reflecting systemic issues in study design, data transparency, and external validation. The lack of standardized imaging protocols and harmonization across different centers further undermines the reproducibility and reliability of radiogenomic models. In this context, external validation is an essential step to pursuit the clinical translation of AI predictive models and should be ideally conducted on large, international, multicenter cohorts. Additionally, prospective studies are exceedingly rare, and external validation is often inadequate, leaving most findings confined to the realm of hypothesis generation rather than practical application. Finally, a major challenge remains the identification of the most relevant radiomic parameters and the establishment of their clinical significance, which continues to limit their integration into routine clinical practice.

A further consideration is that all selected studies relied on single baseline measurements for radiogenomic analysis, limiting the ability to capture tumor heterogeneity and treatment-induced molecular changes over time. A promising yet underexplored approach is Δ-radiomics, which analyzes temporal variations in imaging features to reflect tumor evolution under therapeutic pressure [51]. This method could provide valuable insights, particularly in mutation-driven therapies, where molecular alterations shape treatment response. Integrating Δ-radiomics with longitudinal multi-omics analyses may offer a more comprehensive understanding of tumor dynamics, ultimately enhancing precision oncology.

Future research should address several key areas to advance the clinical utility of PET-based radiogenomics. First, prospective, multicenter studies are essential to enhance the generalizability and robustness of findings; indeed there are some EU projects that have been launched (https://jane-project.eu/project/the-project/ accessed on 13 January 2025) for supporting innovations, such as radiomics. Standardization of imaging protocols, data preprocessing, and genomic analyses is crucial to reduce variability and improve reproducibility. Second, the integration of artificial intelligence and ML approaches could facilitate the development of predictive models with higher accuracy and clinical relevance. These models should be rigorously validated using external datasets and harmonized across different imaging systems to ensure their reliability [52, 53]. Third, the scope of PET-based radiogenomics should be expanded to include underrepresented cancer types, enabling a broader application of this innovative approach, for supporting diverse actionable clinical endpoints, such as treatment response and long-term survival, to demonstrate their real-world impact. Lastly, omic data—genomics, transcriptomics, proteomics, and metabolomics—can drive PET biomarker development by identifying molecular targets linked to tumor biology and treatment response. Genomics and transcriptomics reveal mutated or upregulated genes, guiding PET tracer design for oncogenic pathways. Proteomics aids in developing radiopharmaceuticals targeting overexpressed tumor proteins, while metabolomics identifies unique metabolic signatures, enabling PET imaging of altered metabolism and hypoxia.

## Conclusions

PET-based radiogenomics offers exciting opportunities for advancing precision oncology, though its clinical integration remains hindered by preliminary findings, methodological shortcomings, and limited applicability beyond lung cancer. Addressing these challenges will require a concerted effort to standardize research methodologies, expand the scope of investigation, and bridge the gap between exploratory research and clinical practice. With sustained investment and collaboration across disciplines, radiogenomics has the potential to become a cornerstone of personalized cancer care, providing invaluable insights into tumor biology and improving outcomes for patients worldwide.

## Electronic supplementary material

Below is the link to the electronic supplementary material.

Supplementary Material 1

Supplementary Material 2

## Data Availability

The dataset used and/or analyzed in the current manuscript are available from the corresponding author upon reasonable request.
