# Supplementary material for: Insights into pet-based radiogenomics in oncology: an updated systematic review
Source: Eur J Nucl Med Mol Imaging. 2025 Apr 7;52(11):4184–99. doi: 10.1007/s00259-025-07262-7 (PMC12396984; doi:10.1007/s00259-025-07262-7)
Supplement: Supplementary file 1 — Supplementary Material 1 [file 259_2025_7262_MOESM1_ESM.docx]

| **Table 1S.** CASP assessment of the included studies | | | | | | | | | | | |
| --- | --- | --- | --- | --- | --- | --- | --- | --- | --- | --- | --- |
| **Paper, PMID** | **Q1** | **Q2** | **Q3** | **Q4** | **Q5** | **Q6** | **Q7** | **Q8** | **Q9** | **Q10** | **Appraisal summary** |
| Nair et al.  [31] | Yes | Yes | Yes | Yes | Yes | Can’t tell | No | Yes | Yes | Highly  valuable | Positive |
| Gevaert et al.  [15] | Yes | Yes | Yes | Yes | Yes | Can’t tell | No | Yes | Yes | Highly  valuable | Positive |
| Bakr et al. [29] | Can’t tell | Yes | Yes | Yes | Yes | Can’t tell | No | Yes | Yes | Highly  valuable | Positive |
| Kim et al. [16] | Yes | Yes | Yes | Yes | Yes | Yes | Yes | Yes | Yes | Highly  valuable | Positive |
| Kirienko et al.  [19] | Yes | Yes | Yes | Yes | Yes | Yes | Yes | Yes | Yes | Highly  valuable | Positive |
| Aide et al.  [20] | Yes | Yes | Yes | Yes | Yes | Can’t tell | No | Yes | Yes | Highly  valuable | Positive |
| Chen et al.  [23] | Yes | Yes | Yes | Yes | Yes | Can’t tell | Yes | Yes | Yes | Can’t tell | Positive |
| Ju et al.  [24] | Yes | Yes | Can’t tell | Can’t tell | Yes | Can’t tell | No | Yes | Yes | Can’t tell | Indeterminate |
| Hinzpeter et al.  [30] | Yes | Yes | Yes | Yes | Yes | Yes | Yes | Yes | Yes | Highly  Valuable | Positive |
| Sujit et al.  [28] | Yes | Yes | Yes | Yes | Yes | Can’t tell | Yes | Yes | Yes | Highly valuable | Positive |
| Ning et al.  [27] | Yes | Yes | Yes | Yes | Yes | Can’t tell | Yes | Yes | Yes | Highly  valuable | Positive |
| Kesch et al.  [26] | Yes | Yes | Yes | Yes | Yes | Yes | Yes | No | Yes | Highly  valuable | Indeterminate |
| Ferrer-Lores et al  [22] | Yes | Yes | Yes | Yes | Yes | Yes | Yes | Yes | Yes | Highly  valuable | Indeterminate |
| Kim et al  [25] | Yes | Yes | Yes | Yes | Yes | Can’t tell | Yes | Yes | Yes | Highly  valuable | Positive |
| Lim et al  [18] | Yes | Yes | Yes | Yes | Yes | Can’t tell | Yes | Yes | Yes | Highly  valuable | Positive |
| Choi et al  [32] | Yes | Yes | Yes | Yes | Yes | Can’t tell | No | Yes | Yes | Highly  valuable | Positive |
| Lee et al  [21] | Yes | Yes | Yes | Yes | Yes | Can’t tell | Yes | Yes | Yes | Highly  valuable | Positive |
| Vlachavas et al  [17] | Yes | Yes | Can’t tell | Can’t tell | Yes | Can’t tell | Yes | Can’t tell | Yes | Can’t tell | Positive |

Questions of the CASP assessment: Q1) Was there a clear statement of the aims of the research?; Q2) Is a qualitative methodology appropriate?; Q3) Was the research design appropriate to address the aims of the research?; Q4) Was the recruitment strategy appropriate to the aims of the research?; Q5)Was the data collected in a way that addressed the research issue?; Q6) Has the relationship between researcher and participant been adequately considered?; Q7) Have ethical issues been taken into consideration?: Q8) Was the data analysis sufficiently rigorous?; Q9) Is there a clear statement of findings?; Q10) How valuable is the research?
