# Supplementary material for: Insights into pet-based radiogenomics in oncology: an updated systematic review
Source: Eur J Nucl Med Mol Imaging. 2025 Apr 7;52(11):4184–99. doi: 10.1007/s00259-025-07262-7 (PMC12396984; doi:10.1007/s00259-025-07262-7)
Supplement: Supplementary file 2 — Supplementary Material 2 [file 259_2025_7262_MOESM2_ESM.docx]

| **Table 3.** Summary of machine learning studies’ data mining | | | | | |
| --- | --- | --- | --- | --- | --- |
| **Authors** | **AI SW (class)** | **Data-Mining methods** | **n. of patients in training/test set** | **Validation (n. of patients)** | **Validation Test** |
| Nair et al.  [31] | MATLAB (C),R v.  2.11.1 (OS), SASTM v9.2 (C), IPATM v.9.0 (C) | LR | 25/63 | External (84) | Lasso Cox-proportional  hazards |
| Gevaert et al.  [15] | R v.1.28 (OS), v.2,35-8 (OS) | LR | 25/1 (iterative) | External* | leave-one-out cross validation |
| Bakr et al. [29] | N.A. | N.A. | N.A. | N.A. | N.A. |
| Kim et al. [16] | N.A. | N.A. | N.A. | N.A. | N.A. |
| Kirienko et al.  [19] | RULe eXtractor 4.0 suite (C) | LLM | 52/22 | N.A. | N.A. |
| Aide et al.  [20] | XLSTAT v.2019 (C) | LR | 87/22 | Internal | 100-fold cross validation |
| Chen et al.  [23] | R v.4.3.0 (OS) | LR,, LGR | 135/88 | Internal | N.A. |
| Ju et al.  [24] | R (OS) | RF | 42/10 | Internal | 10-fold cross validation |
| Hinzpeter et al.  [30] | N.A. | APS-LR | 108/20 | Internal | 1000 cross validation |
| Sujit et al.  [28] | R (OS) | Cox regression | 199/133 | External (62) | Pearson’s Chi-square two sides |
| Ning et al.  [27] | Python (OS) | kNN,RF,XGB,SVM,LGR, SHAP, permutation | 102/44 | Internal | 100-fold Monte Carlo cross validation |
| Kesch et al.  [26] | N.A. | N.A. | N.A. | N.A. | N.A. |
| Ferrer-Lores et al  [22] | Python v.3.8.12, R v.4.2.0 and RStudio (OS) | LGR | 33 | N.A. | N.A. |
| Kim et al  [25] | Python (OS)  CNN; Keras 2.3.1 (OS) | RF  GBCNN | 37/15 | N.A. | N.A. |
| Lim et al  [18] | N.A. | N.A. | N.A. | N.A. | N.A. |
| Choi et al  [32] | N.A. | N.A. | N.A. | N.A. | N.A. |
| Lee et al  [21] | R (OS) | GLM,LDA,QDA,kNN,SVM,NN,RF | N.A. | Internal | 5-fold cross validation |
| Vlachavas et al  [17] | R v.3.2.2, 3.3.1 e 3.5.0 (OS) | LR,, RF | N.A. | External* | 10-fold cross validation |

*****only for the genomic component, external public independent dataset

APS-LR= All Possible Subset Logistic Regression; CNN= Convolutional Neural Network; GB= gradient boosting; GLM= Generalized Linear Model ; kNN= k-nearest neighbors; LDA= Linear Discriminant Analysis ; LGR=logistic regression ;LLM=Logic Learning Machine; LR= lasso regression; NA = Not Available ; NN= neural network; QDA= Quadratic Discriminant Analysis ; RF=random forest; SHAP=SHapley Additive exPlanations;; SVM=support vector machine;SW=software; XGB=extreme gradient boosting;
